# DOST: A consolidated health behavior model that maps factors influencing cancer screening uptake

**DOI:** 10.1186/s13690-025-01517-3

**Published:** 2025-03-17

**Authors:** Jyoshma Preema Dsouza, Stephan Van den Broucke

**Affiliations:** 1 Department of Life Sciences and Medicine (DLSM), Faculty of Science, Technology and Medicine (FSTM), University of Luxembourg, Belval, Esch-sur-Alzette,, Luxembourg; 2 Institut de Recherche en Sciences Psychologiques (IPSY), Université Catholique de Louvain, 1348 Ottignies, Louvain-la-Neuve, Belgium

**Keywords:** Cancer screening acceptability, Screening inequities, Models of screening behavior, Health behavior model for cancer screening, Cancer screening barriers

## Abstract

**Background:**

Cancer is a leading cause of death worldwide, particularly in low- and middle-income countries (LMICs), where preventive interventions like screening and vaccination face challenges due to limited resources. Despite the availability of user-friendly screening methods, uptake remains poor. Psychological theories are recommended to identify and address determinants of screening participation; however, existing models often focus on a limited range of domains and overlook critical belief-related factors needed to encourage screening uptake. A comprehensive, integrated model addressing these gaps could significantly improve the identification of barriers to screening.

**Methods:**

This conceptual paper proposes a model that maps potential barriers to cancer screening uptake through the lens of beneficiaries. The ‘Determinants Of Screening upTake’ (DOST) model was systematically developed through a series of steps integrating three existing health behavior theories that have been successfully used previously to improve screening uptake: the Health Belief Model (HBM), the Theory of Planned Behavior (TPB), and the Theory of Care-Seeking Behavior (TCSB).

**Results:**

The DOST model integrates dimensions represented in existing health behavior models, presenting a detailed map of potential barriers in real world, faced by beneficiaries of screening. These barriers are categorized systematically to enhance understanding and facilitate its use among non-experts in empirical research.

**Conclusion:**

By integrating multiple models, the DOST model offers a comprehensive framework that combines theoretical robustness with practical guidelines. It highlights psychosocial barriers that influence screening attitudes, intentions, and uptake. The model can guide the assessment of screening determinants in populations and support the design of educational messages or interventions aimed at increasing screening uptake.

**Table:** 

Text box 1. Contributions to the literature
• **Bridging theory and practice:** This study introduces a conceptual model that integrates behavioral theories into a comprehensive framework to explain barriers to cancer screening. It makes complex theoretical constructs accessible for practical use in empirical research.
• **Identification of Root causes**: The model identifies underlying reasons for the non-uptake of cancer screening. It serves as a foundation for creating survey tools that connect psychological theories with real-world barriers.
• **Contextual insights**: The research highlights the vast range of psychosocial barriers that are theoretically categorized, offering valuable insights into the prioritization of the modifiable ones. The model presented here allows universal applicability and contextual adaptability, crucial for tailoring tools and interventions to specific populations.
• **Contribution to Cancer Screening Research**: The DOST model (Determinants Of Screening upTake) was developed using cervical cancer screening as a reference, given its well-documented barriers and significant public health impact. We propose that the DOST model is adaptable to other cancer screening programs, including breast, colorectal cancer, and prostate cancer which offer similar challenges. However, its application requires careful validation to ensure construct validity in assessment tools. Ongoing research is currently testing this model in a multi-contextual study, including the development of a questionnaire and an educational tool designed around its framework. To prevent misapplication, we recommend awaiting the preliminary validated draft of these tools, which are undergoing testing to ensure they accurately capture the domains the model intends to measure.

## Background

Cervical cancer is the fourth leading cause of cancer among women worldwide. In 2022, nearly 0.5 million women were diagnosed with this disease, and 0.3 million died from cervical cancer. However, early detection of human papillomavirus (HPV) and treatment of patients with precancerous lesions can prevent its progression to cancer. The World Health Organization (WHO) therefore recommends that women undergo tests to screen for precancerous lesions at least once if not regularly every five years [1]. The WHO also recommends specific strategies to improve screening and follow-up, such as the ‘screen and treat’ approach, which is tailored to the context of implementation, particularly in areas where issues related to geographical accessibility persist [2]. The ‘screen and treat’ approach involves immediate treatment following positive screening results to prevent disease progression, making it especially suitable for resource-constrained settings.

Despite these recommendations, the uptake of screening for cervical cancer remains low, particularly in low- and middle-income countries (LMICs), where the disease burden is significant [3]. This issue can partially be attributed to organizational limitations within the health system—in resource-poor settings—which pose substantial challenges to providing organized cervical cancer screening. Moreover, even when well-tested strategies are implemented, they do not always result in successful utilization of screening services because of barriers directly experienced by women. While ensuring the availability, affordability, and accessibility of screening services is critical, poor acceptability or utilization can lead to program failure. Therefore, it is crucial to identify and mitigate the barriers to enhancing women’s use of available screening services.

An individual’s decision to undergo screening, when screening is available, depends on various factors or determinants. These determinants vary by age, culture, time, geography, and physical, psychological, social, and spiritual conditions. Each of these domains includes factors that may either facilitate screening uptake or represent a barrier to uptake by the individuals or beneficiaries (i.e., individuals who are targeted for screening programs based on specific criteria such as age, risk factors, or potential benefits from early detection and preventive care). Because any of these factors could influence a woman’s decision to undergo screening, it is important to account for all of them. To that effect it is useful to utilize a model that captures all determinants and classifies them logically for easy application, thus capturing a large range of determinants. Yet, while numerous studies have used existing models to explore or address cervical screening uptake [4–9], no single model has emerged as one that is simplified for practical use and encompasses all potential reasons for the non-uptake of screening. The proposed **DOST** model, developed with cervical cancer screening as a reference, provides a comprehensive framework to identify barriers across individual, interpersonal, societal, and organizational levels, and is applicable in diverse screening contexts and populations.

### Unveiling determinants of screening uptake– modifiable and nonmodifiable barriers

The socioecological model developed on Bronfenbrenner ‘Ecologic Systems Theory’ [10] provides a structured framework to provide an overview of various determinants of screening uptake by categorizing them across different levels of social interaction: individual, interpersonal, sociocultural, health system, and political. It allows us to differentiate between external factors that constitute the health system and political factors (those that exist outside of the individual or the beneficiary) and those that are related to beneficiaries themselves (Beneficiary: Individuals targeted by screening programs based on specific criteria such as age, risk factors, or potential benefits from early detection and preventive care).

Before exploring the reasons for the non-uptake of cancer screening, it is essential to recognize that each individual may face unique barriers and facilitators influencing their decision to engage in screening. Effectively addressing these issues requires categorizing and simplifying them to focus on those particularly susceptible to change, which could significantly improve ‘attitudes’ toward screening and eventually action, i.e., screening. Psychosocial barriers are crucial in this context, as they directly inform the decision-making process and utilization of screening services. These barriers are of particular interest in research because of their modifiable nature. ‘Modifiable factors’ include beliefs such as sociocultural beliefs, individual perceptions, beliefs influenced by partners or family members, and spiritual influences. These factors are considered modifiable because they can be altered. For example, in the context of applying theories to enhance or promote screening uptake, substantial opportunities exist to mitigate these ‘modifiable barriers’ through intervention design or educational messages during health programs, awareness campaigns, or implementation studies, as opposed to ‘nonmodifiable’ factors such as age or financial status, making it especially relevant in public health research and programs. Given the variability in the importance of each factor among individuals, it is prudent to anticipate and comprehensively address all modifiable factors to increase screening uptake. Therefore, a model that addresses all modifiable factors, both at the individual and contextual levels, is essential. A holistic approach that considers and tackles both psychosocial and logistical barriers is critical for developing interventions that are meaningful and effective in enhancing screening uptake.

Having established the importance of addressing modifiable factors, health behavior theory-based research has extensively utilized theoretical models for three primary purposes: (a) exploring determinants, including barriers and facilitators, of screening uptake [4, 11–13] or *‘exploratory purpose’*; (b) understanding the mechanisms of behavior change and the interrelationships among determinants [14]; and (c) informing interventions designed to promote screening uptake [15, 16] or ‘*intervention purpose’* [17–19]. Health behavior models are particularly suitable for explaining the psychological processes involved in decision-making related to screening, which are based on beliefs, perceptions, and emotions [20]. Since these theories are broadly applicable, they have been widely utilized and recommended for translational implementation research. Most importantly, these models are effectively used to investigate or address psychosocial determinants influencing screening uptake [15]. This paper introduces a conceptual model, the DOST (determinants of screening uptake) model, that maps the psychosocial determinants of screening uptake, which is grounded in health behavior theories. Additionally, recognizing the importance of external factors, i.e., the health system and policy factors, this model provides a clear distinction of barriers across the domains of the socioecological model.

### Existing models and frameworks

The development of health behavior models has significantly shaped our understanding of barriers and facilitators to cancer screening uptake. Traditional models, such as the Health Belief Model (HBM) and the Theory of Planned Behavior (TPB), have guided research and interventions aimed at understanding and improving screening behaviors [12, 15, 21], but also show a number of important limitations.

#### Traditional Health Behavior models

*The Health Belief Model* (HBM) [45] was created by the social scientists in the early 1950s to understand why people did not use screening tests for early disease diagnosis. According to the HBM, a person’s likelihood of adopting a health behavior can be predicted by how they perceive the disease severity, their risk of contracting an illness or disease, how much they believe the recommended health behavior or activity works and the confidence in their ability to perfrom the behavior despite potential barriers.

The *Theory of Planned Behavior* (TPB) [46] is an adaptation and extension of the (older) *theory of reasoned action* and is often used in health research to explain intentions to perform health behavior, which is assumed to be the main predictor of actual behavior. It suggests that an individual is likely to exhibit a certain behavior if they have positive intentions to do so, and the intention depends on their belief that the behavior will give a positive outcome; they have the ability to do it, whereas a significant other will support them in the adoption of this behavior that they perceive to be doable.

The HBM and TPB have been instrumental in identifying individual-level determinants, such as perceived susceptibility, self-efficacy, and health motivation. These models offer valuable insights into understanding why individuals may or may not engage in cancer screening. However, their contributions are primarily limited to explaining behavioral intentions, leaving a significant portion of variance unexplained. They fail to address systemic and contextual factors, such as affordability and accessibility, which are critical in low- and middle-income contexts. Among the main criticisms and limitations of these models are: (1) the fact that they focus narrowly on psychological determinants without considering the interplay between individual and external factors (e.g., cultural norms, health system constraints, etc.); and (2) the fact that although each model explains part of the variance in behavioral intentions and, to a lesser extent, behavior, a significant portion of the variance remains unexplained [22].

### Integrated models

The limitations of traditional models to address multifaceted barriers, particularly in complex settings, has prompted researchers to integrate theoretical domains into more comprehensive frameworks [23]. These integrated frameworks combine theoretical domains to provide a more complete and holistic understanding of health behavior. For examples, the *psychosocial determinants of socioeconomic inequalities in the cancer screening participation* framework [24] looks at the interaction between psychological and structural barriers and explain why individuals from poor socioeconomic backgrounds perceive greater barriers to screening uptake than do those living in less deprived situations. Similarly, the *Integrative Model* [13] assesses factors affecting HPV vaccine acceptance [25] by integrating the domains of the HBM and TPB theoretical models. The *Integrated Behavioral Model (IBM)* [26] combines constructs such as knowledge, salience, and environmental constraints with existing theoretical domains to provide a more comprehensive understanding of screening behavior determinants and has been used to explore cervical screening barriers [27] or reduce cancer screening delay [28]. The *COM-B Model* [29] and the *Behavior Change Wheel* (BCW) [30] combine capability, opportunity, and motivation with intervention strategies and policy considerations, addressing behavioral and contextual barriers in a systematic way. The *Theoretical Domains Framework (TDF)* [31] further expands on the psychological and organizational determinants of behavior, and can be used in combination to facilitate a systematic exploration of barriers to and facilitators of health behaviors such as cervical screening [32]. Finally, the *Integrated Screening Action Model* (I-SAM) [33] amalgamates pertinent health behavior theories to elucidate cancer screening behavior, with a primary emphasis on stages of behavior change and the influence of individual and interpersonal levels across these stages. It integrates these factors to provide a comprehensive framework for understanding the end variables that explain cancer screening uptake.

For a detailed review of these traditional and integrated models, their theoretical underpinnings, their applications to cervical cancer screening, their contributions to improving cancer screening uptake, and their limitations, the reader is referred to the author’s chapter on application of health behavior theories [34]. There are indeed various challenges to developing or applying these models [35]. According to Hein, ‘*Integration is more than simply combining theories. Combining theories implies the use of a simple additive model*,* while integration implies critical testing of these constructs, and constructs will be added only if they have supplementary value theoretically and empirically and will lead to the development and testing of new hypotheses*’ [35]. In the domain of understanding cancer screening, the integration or combination of health behavior theories for both research and practical applications has provided significant insights into how models can be combined or integrated for various purposes.

### Addressing the theoretical and practical gaps

While traditional models such as the HBM and the TPB often fail to incorporate a number of important behavioral, particularly external factors such as health system or political factors, integrated models such as the capability, opportunity, motivation, and behavior (COM-B) model, the behavior change wheel (BCW), and integrated screening action model (I-SAM) include both psychosocial and external factors and are widely cited. However, the use of these models to map, explore, or identify determinants of cancer screening uptake remains problematic because they do not provide an exhaustive list of the root causes, such as poor knowledge and false beliefs existing in real-world phenomena. While more detailed exploration of the underlying knowledge, awareness and beliefs is typically relegated to empirical research involving qualitative interviews or focus groups that offer rich insights into their lived experiences, this method can be time-consuming, and data may not be representative owing to its small sample size. In such a case, a top-down approach can be useful and a model mapping these factors can guide systematic exploration or mitigation. Such a model will extend the traditional and integrated models by systematically addressing these root causes, or the ‘belief-related factors’ that needs to be mitigated to influence perception and motivation to undergo screening.

While a theory-informed approach can be helpful to explore determinants of screening uptake, a comprehensive model that can serve as a guide or checklist for designing questionnaires to explore them or inform educational messages for awareness campaigns to screen uptake is thus far lacking, which makes it challenging to assess or mitigate these factors comprehensively. A lack of clarity on the underlying factors due to a lack of a comprehensive model can also lead to inconsistent findings in the exploration of these belief-related barriers. Most studies utilize the domains of existing traditional models to select or develop tools, questionnaires, or educational interventions, but there is often ambiguity in how these elements should be developed. This raises questions as to which model should be used, how many items are needed, which specific domains should be prioritized, and how questions should be worded to operationalize the psycho-social barriers. For example, domains such as the ‘Perceived Severity’ or ‘Perceived Benefit’ of the HBM, have been measured in various ways using different assessment tools [15]. This ambiguity among researchers who try to apply such models can stem from a lack of understanding of the models or their limitation to bring types of barriers into broader categories. Therefore, a model with a simplified list of belief-related barriers or determinants can make it less ambiguous to formulate questionnaires to explore them in formative research.

This problem can also affect translational intervention studies aiming to promote or improve screening uptake. If the root causes (e.g., beliefs) are not all targeted by educational or informative interventions, the outcomes may be less effective because certain determinants remain unaddressed.

The complexity and interdependence of barriers to screening uptake mean that if any single barrier is overlooked, the intervention may inadvertently yield poor outcomes or no improvement in screening attitudes or beliefs, despite the educational program. This can result in a Type III error [36] in intervention studies trying to improve screening uptake. A Type III error occurs when the intervention is deemed ineffective in improving screening uptake, not because it addresses the wrong problem but because it misses a critical component of the correct problem. It thus might appear ineffective or even counterproductive because it failed to address a significant aspect that was not considered. To avoid this, applying the ‘universal precautions’ principle is ideal, although challenging. This principle, originally from healthcare [37], when applied in this context, suggests that we should assume all potential barriers are present and take comprehensive steps to address them. This approach ensures a thorough and holistic intervention strategy, enhancing the likelihood of improving knowledge in all areas related to disease and screening. Therefore, a model that highlights belief-related barriers could guide educational interventions to formulate messages necessary to target these barriers.

Comprehensive models such as TDF comprehensively capture several domains of determinants, and can thus be relevant for categorizing barriers and facilitators for screening [38] across broad categories. But they can be operationally difficult to explore specific belief-related factors [39, 40]. As they contain many constructs, some of which overlap, it can be challenging to select relevant domains and constructs for the purpose of mapping determinants of screening, making the framework less user friendly for implementers who don’t have a good theoretical background [41].

There is a pressing need for simplified yet comprehensive models that not only encompass a broad range of determinants but also facilitate the creation of tools or questionnaires to assess beliefs directly and easily. Such model could not only explore beliefs preventing screening uptake but also inform development of educational messages to enhance knowledge, target or modify the deep-rooted beliefs, and motivate the individual to uptake screening. These integrative efforts are vital for advancing public health strategies, especially for nonexperts, to improve outcomes in preventable cancers like cervical cancer screening and others.

### Bridging gaps with the DOST model: enhancing practicality and theoretical depth to map determinants of screening uptake

A recommended approach to modifying screening beliefs involves the use of psychological health behavior theories to elucidate these beliefs. A more effective strategy is to integrate the traditional health behavior theories [35, 42], considering the broad range of social determinants i.e., influence of family, society, etc [43], that influence these beliefs.

The DOST (Determinants Of Screening upTake) model builds on and extends existing health behavior theories by focusing on and simplifying belief-related factors, representing real-life barriers to screening acceptance, making it easier to explore these barriers. Educational interventions play a vital role in addressing these barriers by framing specific informative messages to mitigate knowledge- and belief-related obstacles. The DOST model complements existing theoretical models by providing a practical framework that targets these belief-related barriers, ensuring that educational interventions are well-targeted and successful.

### Brief overview-DOST

The ‘DOST-Determinants Of Screening upTake’ model was developed to build on the strengths of both traditional and integrated models while addressing their limitations and represents an innovative approach in cancer prevention health behavior research and is uniquely designed to extend and bridge both theoretical and practical gaps in existing models. It can be used to explore reasons for the non-uptake of cancer screening or to promote screening via educational messages. By building on and synergistically combining traditional theories such as the HBM, the TPB, and the Theory of Care Seeking Behavior (TCSB) [44], it accommodates a full spectrum of psychosocial determinants across meaningful theoretical domains because psychosocial barriers play a significant role in decision making in screening utilization. The combinations of the variables from different models are tested primarily to understand the explained variance of integration on screening intention. Additionally, DOST employs socio-ecological model categories to systematically map and display determinants of screening uptake. These include individual-related factors and external influences, such as interpersonal and sociocultural beliefs (e.g., opinions of loved ones that impact individual decisions). Structural barriers, including limited screening facilities, insufficient manpower, inadequate policies, and high screening costs, are also considered.

A detailed explanation of these factors is provided in the description of the model. Crucially, the model is grounded in empirical evidence, ensuring that its subdomains accurately reflect the actual psycho-social determinants, or ‘beliefs,’ that explain its domains. These subdomains, representative of real-life barriers, facilitate the development of targeted questions for empirical research, thereby simplifying the application of the model for implementers and program managers who may not be psychology experts. By categorizing these barriers under traditional model domains and including external factors, the DOST model complements and enhances existing health behavior theories and socio-ecological model, offering a holistic view of the determinants influencing cancer screening behavior, and deeper focus on belief related factors influencing screening acceptability relevant for studies focusing on these factors.

## Method

To develop a consolidated model, we followed a systematic stepwise approach consisting of four consecutive steps: (1) Step 1: A systematic review to identify the health behavior theories that explain screening behavior (2). Step 2: Evaluating Predictive Strength of Health Behavior Models for Cervical Cancer Screening Intention (3). Step 3: Integration of the identified health behavior theories and domains into a conceptual model and testing its ability to predict cervical cancer screening intention (4). Step 4: Mapping the domains that may influence screening uptake or intention (components from the integrated health behavior model) across the levels of the socioecological model, comparing them with real-world barriers identified through emperical qualitative and quantitative studies.

### Setting

Empirical research related to model testing was conducted in a low-middle-income country (India) selected to represent a setting characterized by significant challenges faced by women and the healthcare system. This choice aimed to address cultural and belief barriers, which are presumed to be pronounced in such contexts.

### Step 1: A systematic review to identify the health behavior theories that explain screening behavior and intention

The initial step in our approach to developing the integrated DOST model was a systematic review, the results of which have been published elsewhere [15]. The objective of this review was to evaluate existing health behavior theories or models that are extensively employed to assess or predict barriers to cervical cancer screening or those utilized to inform educational interventions. This was done to ensure that the final model was built upon relevant and empirically supported theoretical foundations. The results of the study aided in pinpointing the most pertinent and effective frameworks and strategies, guiding the development and refinement of the DOST model.

Many studies have also explored the utilization of health behavior theories to explain breast cancer screening behavior [12]. Our review enabled to extract data on health behavior models used in assessing cervical cancer screening barriers and in interventions that could mitigate barriers to screening uptake and promote screening. It confirmed that health behavior theories are crucial in clarifying intentions and behaviors related to cervical cancer screening and in developing interventions to enhance screening uptake, and that interventions that encompass all theoretical constructs display superior effectiveness in transforming perceptions and amplifying the adoption of cervical cancer screening. However, it also revealed that the application of health behavior theories, though advantageous, has been inconsistent, in that the HBM was primarily used to interpret behaviors, whereas the TPB was more effective in explaining screening intentions.

### Step 2: Evaluating predictive strength of health behavior models for cervical cancer screening intention

Systematic reviews of existing health behavior models have revealed that two methods have been widely applied to cancer screening (i.e., the HBM and the TPB). A third model, the theory of care-seeking behavior(TCSB) [44], is less widely used but has components that significantly influence screening behavior. Lauver’s Theory of Care-Seeking Behavior (TCSB), developed in 1993 and based on Triandis’ theory of general behavior, posits that individuals are more likely to engage in a behavior if they adhere to healthy practices, experience positive emotions, and perceive support from significant others, alongside favorable sociodemographic conditions. In the context of cancer screening behavior, the inclusion of the “Affect” domain within the conceptual model is pivotal because of its significant influence on various other domains, such as perceived susceptibility and perceived benefits.

In cervical cancer screening, for example, the domain of “Affect” is important because it encapsulates emotions such as fear, anxiety, or embarrassment. These emotional responses can significantly impact other domains within the model; for instance, poor knowledge and false beliefs can directly lead to fear or anxiety, which may lead to heightened perceived susceptibility or diminished perceived benefits. Such emotional responses often stem from inadequate knowledge, awareness, false beliefs or even a lack of social support, necessitating specialized attention and empathy toward individuals experiencing these emotions. Consequently, addressing these emotional factors becomes imperative in designing effective interventions to promote cancer screening. The incorporation of the ‘affect’ domain into the conceptual model enhances our ability to understand cancer screening behavior from an emotional perspective. By including emotions such as fear, anxiety, and embarrassment, the model acknowledges the significant role these feelings play alongside cognitive determinants. This comprehensive approach allows us to understand the reasons for the non-uptake of screening not only through a cognitive lens but also through the emotional experiences of individuals. Furthermore, to promote screening uptake, interventions can be designed to alleviate fear and anxiety by providing clear, empathetic communication and supportive counseling. This dual focus on emotions and cognition makes the model more effective and applicable.

### Step 3: Combine the domains or components of the most predictive health behavior models into a conceptual model and test the ability of the integrated model to predict cervical cancer screening intention

To identify which model can predict screening intention better, a questionnaire study, which is published elsewhere, was undertaken to test the ability of each of these models to explain screening intention [47]. While it was found that the TPB and the HBM could significantly predict screening intention, certain components of the theory of care seeking behavior, such as ‘habits’, were significantly associated with the intention to be screened [47]. Therefore, the components of these three models were combined and tested for their combined validity to explain ‘screening intention’ which is considered a universal predictor of screening behavior. The integrated model performed better in explaining screening intentions than each of the individual models did [14].

### Step 4: Mapping the factors that may influence screening uptake (components from the integrated health behavior model) across the levels of the socioecological model

To map the potential factors that explain screening uptake, the domains or constructs of the TPB, the HBM and the TCSB were integrated into a consolidated model that includes all potential psychological factors that significantly influence cancer screening intention.

**Fig. 1:**
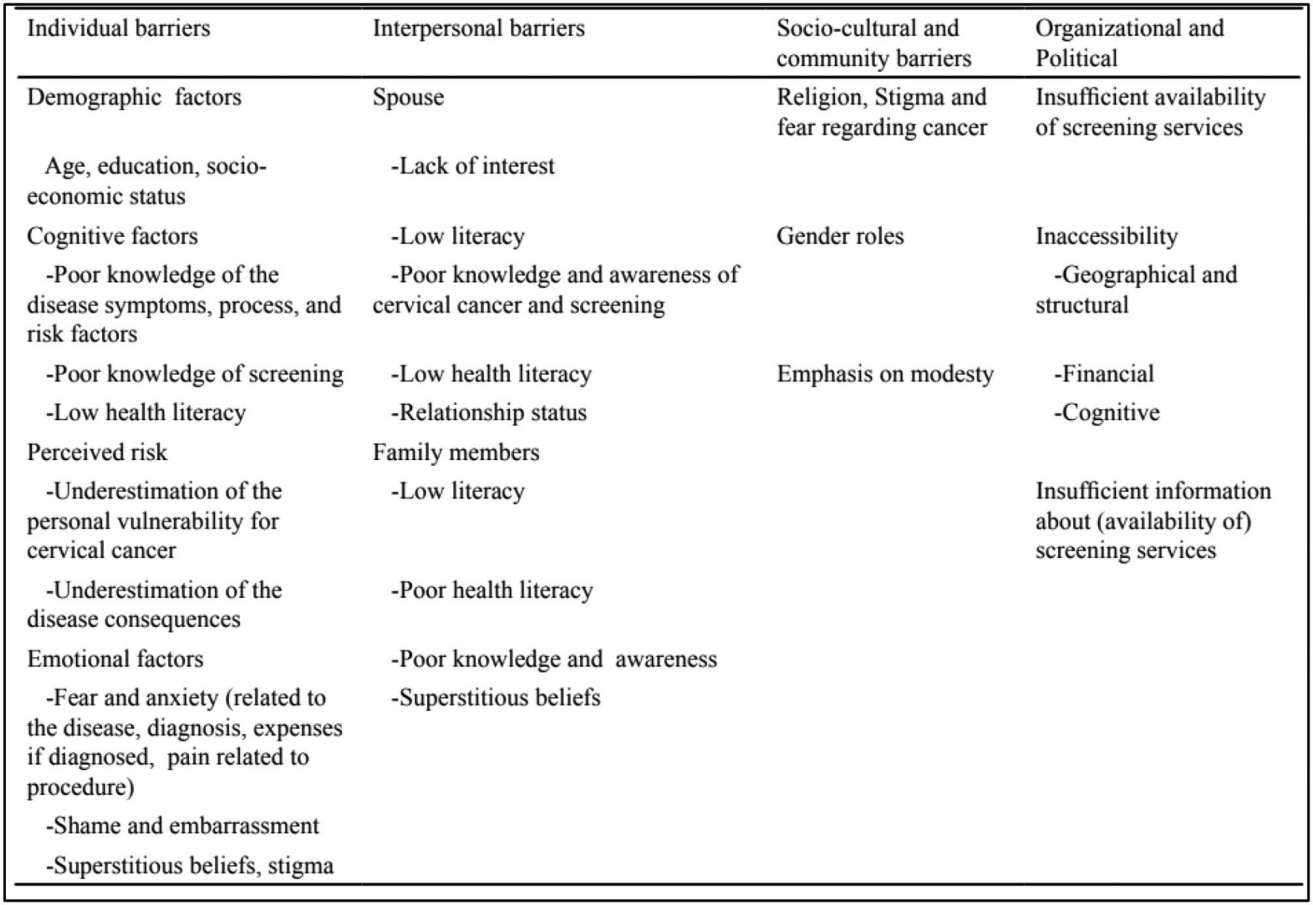
Barriers to screening uptake across socioecological levels [50]

The systems model of behavior change suggests the consideration of external factors that influence behavior [48]. To employ the systems approach, we used the socioecological model proposed by Bronfenbrenner [49], which shows that factors at various levels of social interaction can act as facilitators or barriers to behavior. When applied to health behavior, various levels of an individual’s social interaction factors can influence a person’s decision to participate in screening: individual factors (e.g., sociodemographic profile, knowledge and awareness, beliefs, self-perceptions), interpersonal factors (e.g., significant others that might influence one’s beliefs), sociocultural factors (e.g., influences of the community, culture), and organizational factors (e.g., availability of screening services, health programs, health coverage). The factors that may influence cervical cancer screening could be displayed across the socioecological model, as shown in Fig. 1, which is published elsewhere [50]. While the initial mapping and validation were focused on cervical cancer screening, the socioecological structure ensures broader applicability. For instance, similar psychosocial barriers—such as fear of invasive procedures, embarrassment, or cultural sensitivities—are also observed in breast and colorectal cancer screening. This gives the model scope to be applied or adapted for identifying determinants in other cancer screenings, such as breast, colorectal, or even prostate cancer.

**Fig. 2:**
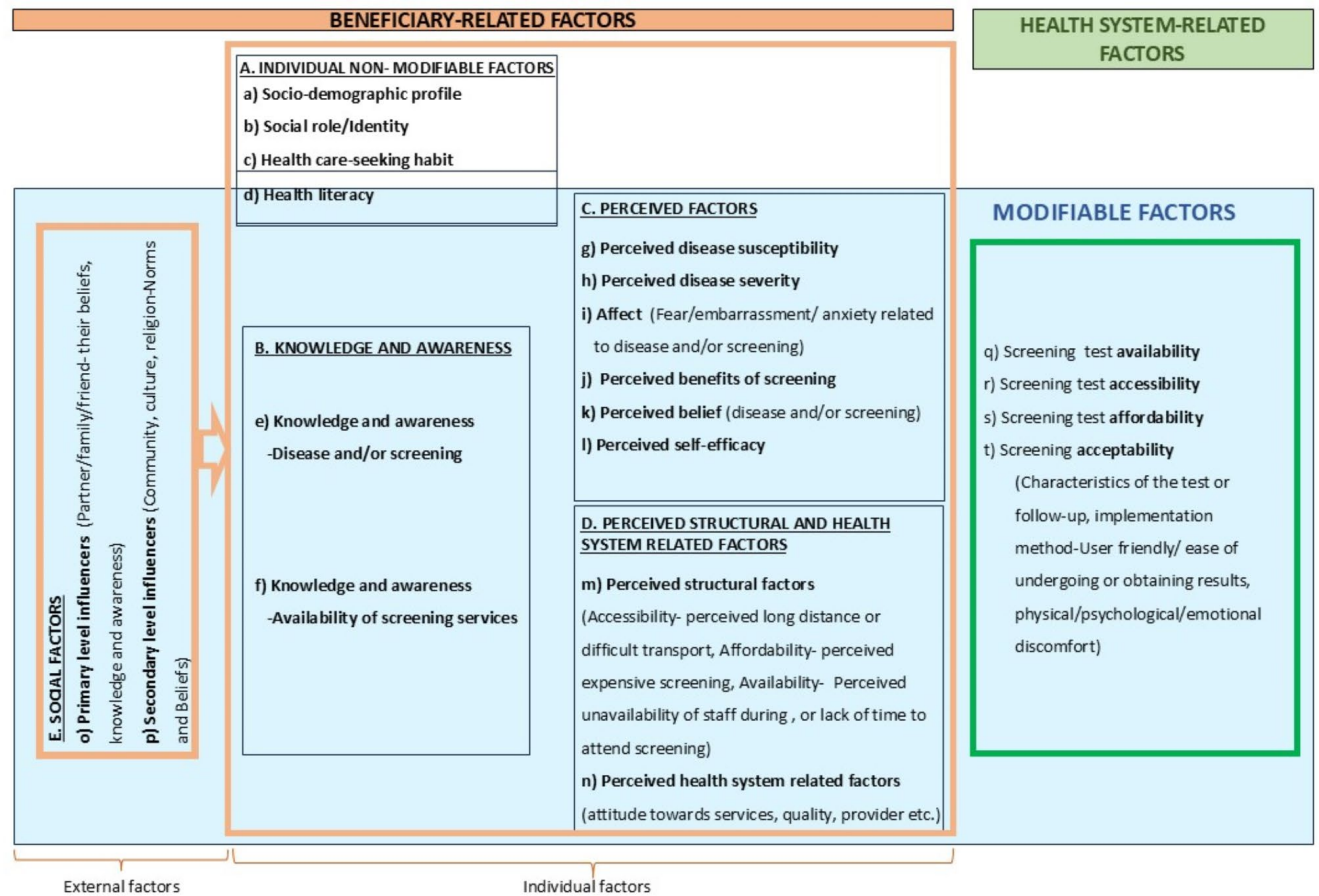
‘*DOST’*– ‘Determinants Of Screening upTake’ model

We added the domain of ‘health literacy’ to our model (seen under the ‘individual factor’ in Fig. 2) because it is a critical concept in public health [51], and several studies have shown its significance in screening behavior [52]. *Health literacy* (HL), is a person’s ability to obtain, understand, appraise and apply information that is needed to make informed health decisions and low health literacy, is associated with poor self-care, poor adherence to medication, less uptake of preventive or health promotional services, and high expenditure on medications [51, 53, 54]. There is evidence in the literature that individuals with low health literacy are more likely to hold fatalistic cancer prevention beliefs [55, 56] or not seek medical care despite experiencing cancer-related symptoms [57] than those with higher health literacy. Our test of the relationship between HL and cervical cancer screening behavior confirmed the importance of HL for cervical cancer screening intentions [14, 58].

In the context of the DOST model, health literacy (HL) plays a crucial role in promoting cervical cancer screening. Although health literacy cannot be directly modified to improve screening, it can be improved from a young age [59], which is why it overlaps with the modifiable and nonmodifiable barriers in our DOST model. Importantly, one’s health literacy level should be considered during screening promotion, and certain considerations can be made during the development of these educational interventions to ensure that individuals with lower health literacy levels are effectively reached and engaged. Additionally, with health psychology expert opinions, we retained the ‘Affect’ domain from the TCSB in the model to be integrated into DOST to highlight the importance of emotional factors that significantly influence the decision to screen.

### Reporting standards

This manuscript adheres to the TIDieR (Template for Intervention Description and Replication) checklist for transparent and comprehensive reporting of the DOST model.

## Results

### The ‘DOST’- determinants of screening-uptake model

Model overview: The DOST model categorizes the factors that may influence screening participation across domains and their components for the ease of understanding and use of its users, who could be program implementers, researchers, etc. The DOST model and domains are displayed in Fig. 2, and their components are displayed in Table 1.

**Table 1:** ‘DOST’ model domains and components

Beneficiary-Related Factors		
A. INDIVIDUAL NON-MODIFIABLE FACTORS		
	***Domains***	***Components***
a	Sociodemographic profile	Education, physical status (presence or absence of warning signs) and cognitive skills, financial status, health insurance status
b	Social role/identity	1. Role in family/community (educator/leader)
		2. Role as social/financial provider
		3. Role as a decision maker for health care seeking, treatment/diagnosis expenses.
c	Health care-seeking habit	1. Individuals’ health care-seeking habit, that reflects their usual care-seeking behavior promptly. (i) check-ups when one has symptoms and (ii) Routine check-ups irrespective of symptoms (asymptomatic screening)
d	Health Literacy	Individual’s ability to access, understand, appraise, and apply the information during the life course for health care, disease prevention or health promotion
B. KNOWLEDGE AND AWARENESS		
	***Domains***	***Components***
e	Knowledge and awareness (disease and/or screening)	1. Knowledge about the disease: Disease etiology, risk factors, clinical features, warning signs
		2. Knowledge about screening: awareness about screening, tests (Ex: What are the tests), methods (Ex: How is screening done), immediate outcomes (Ex: What happens when screening report is positive or negative) and follow-up (Ex: What needs to be done post screening)
f	Awareness of availability (cost, time, type)	Awareness about the location where screening is available, awareness of cost, time and type of screening services that are available.
C. PERCEIVED FACTORS		
	***Domains***	***Components***
g	Perceived disease susceptibility	Perceived susceptibility refers to beliefs about the likelihood of getting a disease or condition (Ex: If the individual thinks she can get the disease or is more susceptible to getting the disease, is more likely to get herself checked for the disease)
h	Perceived disease severity	Perceptions about the seriousness of contracting an illness or about the consequences of leaving it untreated which includes evaluations of both medical and clinical consequences (Ex: If the individual thinks the consequences of the disease will be severe, she is likely to get herself checked) 1. Perceptions on physical consequences of the disease (Ex: Believing that disease can cause death, disability, and pain) 2. Perceptions on psychological consequences of the disease (Ex: Believing that disease can bring sorrow, and worry) 3. Perceptions on social consequences of the disease (Ex: Believing that disease will affect his/her relationship with partner, family or relatives altering the dynamics of the relationship) 4. Perceptions on financial consequences (Ex: Believing that disease can be expensive to treat or manage) 5. Spiritual consequences (Ex: Believing that disease can affect her spiritual faith)
i	Affect	Feelings (Fear, embarrassment, anxiety) caused by ‘Beliefs’ either related to Disease or related to screening.
		1. Affect related to beliefs on nature or characteristics of the disease (Ex: False Beliefs like cancer is communicable or untreatable can cause anxiety and fear. The belief that disease is untreatable can cause fear and demotivate oneself to get checked. Fear of death due to the untreatable nature of the disease or anxiety associated with its poor prognosis. 2. Affect related to belief on cause or risk factors for disease. (Ex: False beliefs like the wrath of God as the cause can induce anxiety or guilt. A belief that reproductive tract diseases are associated with promiscuity can lead to embarrassment) 3. Affect related to one’s beliefs on disease outcomes. (i) Physical outcome on health: Fear of pain/fear of changes or loss of body parts due to the disease/embarrassment related to the body part affected. (ii) Psychological outcome (Ex: Fear of being emotionally dependent on others) (iii) Social outcome (Ex: Fear of being abandoned by family or being a burden to family) (iv) Spiritual (Ex: Shame related to the belief that cancer is caused by punishment from God) (v) Financial outcome: fear of expenditure for cancer treatment
		4. Affect related to performing or undergoing screening (Fear of pain, anxiety and embarrassment related to the test) and its outcome (Ex: anxiety or fear related to positive outcome) It also encompasses fear, anxiety, or embarrassment related to the person conducting the screening or the setting in which the screening takes place.
j	Perceived benefits	Perceptions associated with screening outcomes based on knowledge, experience, or beliefs. 1. Perceived benefit to health (Ex: Screening will prevent disease) 2. Perceived benefit to psychological well-being (Ex: Getting screened will cause me less worry because if diagnosed early it is treatable) 3. Perceived benefit to social relations (Ex: Getting screened can help with early treatment so I can be back to health with my family) 4. Perceived benefit to spiritual well-being (Ex: Getting screened will strengthen my spiritual beliefs) 5. Perceived benefit to financial well-being (Ex: Screening expense is less than the disease treatment-related expense)
k	Perceived barriers (False beliefs)	Perceived tangible and psychological costs of undergoing cancer screening due to beliefs and attitudes.
		Thoughts/cognitions related to disease or screening
		1. Thoughts associated with the disease can prevent screening uptake. (i) Thoughts/cognitions related to disease etiology (Ex: Belief that disease is caused due to one’s sins and suffering leads to repentance), risk factors, nature/characteristics (Ex: Belief that disease is cause due to promiscuity can prevent one from consulting a doctor), warning signs (Ex: Belief that irregular menstruation is normal), management and treatment (Belief that the disease has treatment and good prognosis) (ii) Thoughts/cognitions related to the outcome/impact of disease. (Ex: Outcome of disease on one’s physical health, mental health, social, spiritual, or financial health)
		2. Thoughts associated with screening that can prevent screening uptake. (i) Thoughts/cognitions related to screening: Related to the need for screening (Ex: screening is done only when symptoms occur), screening process or method (Ex: Screening test can be invasive or time consuming)
		3. Thoughts/cognitions related to the outcome or impact of screening can prevent screening uptake. (i) Thoughts of the Outcome of screening on one’s physical health (Ex: Screening can lead to health problems), mental health (Ex: Screening can lead to unnecessary worry if the outcome is positive), social (Ex: Screening can lead to people disliking me), spiritual (Ex: Disease is given by god and one must accept it and not prevent it) or financial condition (Ex: Screening can lead to unnecessary expenses)
l	Perceived Self -efficacy	A strong belief that one is capable of seeking and undergoing screening tests despite existing difficulties or challenges can influence screening uptake
D. PERCEIVED STRUCTURAL AND HEALTH SYSTEM-RELATED FACTORS		
	***Domains***	***Components***
m	Structural barriers	Distance, Time, and cost related to obtaining screening test.
		Perception: Poor accessibility to screening center- Geographical (Ex: long distance, poor transport), Time (Ex: Time-consuming process, long waiting hours, long travel time), Cost (Ex: expensive screening test, expensive transport to screening test, loss of wages per day spent at the screening center)
n	Health system related (perceived factors related to service delivery)	1. Beliefs about the bad attitude of health care providers, and poor quality of care at the health facility, based on information or due to bad experiences in the past can prevent the individual from undergoing screening. 2. Affect (fear/embarrassment/anxiety) related to negative beliefs about the health system, Affect. (fear/embarrassment/anxiety) related to finding or navigating the health system or approaching health professionals.
E. SOCIAL FACTORS		
	***Domains***	***Components***
o	Primary level influencers	Influence of significant people- partner family, friends etc. (Ex: their knowledge and awareness included in ‘B’, Perceived factors included in ‘C’ and perceived structural or health system factors included in ‘D’.
p	Secondary level influencers	Influence of significant community, culture, religion, and tradition (Ex: Knowledge and awareness (B), Perceived factors (B), Perceived health-system factors of people who have common geography, faith, tradition, or culture)
HEALTH SYSTEM RELATED FACTORS		
	***Domains***	***Components***
q	Screening availability	Factors influencing screening availability (Examples: Screening centers are predominantly located in urban areas, leaving rural populations with limited access; Irregular operating hours of screening centers prevent individuals from accessing services at convenient times; shortage of qualified healthcare professionals limits the availability of screening appointments, leading to long waiting times)
r	Screening accessibility	Factors influencing accessibility of Screening Services Geographical Factors (Example: Inability to ensure screening centers are within a reasonable distance for rural or remote populations, or poor transportation infrastructure that hampers individuals’ ability to reach the screening centers) Time related factors (Example: Screening processes that are overly time-consuming, deterring individuals due to long waiting hours and extensive travel time or Inability to offer screening services at times that accommodate individuals’ work schedules, leading to delays) Cost related factors (Example: High costs associated with screening tests that are not subsidized, making them unaffordable for many individuals; Expensive transportation options exacerbate the financial burden of accessing screening services; The economic impact of losing wages due to time spent at screening centers remains unaddressed)
s	Screening affordability	Factors influencing Screening Services’ Affordability (Example: Inability to provide insurance coverage for screening tests, resulting in significant out-of-pocket expenses; Additional costs for follow-up consultations or tests further strain the financial resources of individuals; Lack of financial assistance or subsidized programs for low-income populations limits access to essential screening services)
t	Screening test characteristics/acceptability	Screening test characteristics that influence the acceptability of the test (Example: Inability to develop non-invasive and comfortable screening tests leads to reluctance among potential participants; Screening Methods/algorithms or complex implementation strategies that are confusing or time-consuming deter participation due to the inconvenience they pose; The delay in obtaining screening test results, requiring multiple visits, discourages consistent follow-up)

In the model, barriers to screening are broadly classified as ‘health system-related barriers’, which are challenges faced by the health system to provide screening services (in green), and ‘beneficiary-related barriers’, which are the barriers faced by beneficiaries to utilize the services (in yellow). Theoretically, all the barriers need to be mitigated to promote decision making, which could be a challenging task in practice. However, when broken down into components, it becomes easier to address these separately, especially those that can be mitigated (modifiable barriers).

#### Domains and components of the DOST model

### Health system factors

Cancer screening services or programs may not be implemented systematically leading to inequities. These inequities are due to a vast array of factors influencing implementation of cancer screening programs, which can be categorized as ‘health system-related factors’ (Displayed in green box in the model-Fig. 2). Screening utilization depends on the assurance of screening availability; addressing psychological barriers alone may not be impactful if screening services are not available or accessible, and hence, they must also be considered. Technically, it is essential to preliminarily assess the availability of screening when designing interventions to promote screening uptake. Neglecting to ensure the availability, affordability, accessibility and acceptability (Components ‘q’, ‘r’, ‘s’ and ‘t’ in DOST model) of screening could lead to incongruous and counterproductive interventions. The ‘health system barriers’ must be addressed foremost. This will enable a focus on beneficiary-related barriers to screening uptake. Typically, health system factors listed in Table 1, are more pronounced in LMICs because of limited health system resources [60]. While frameworks exist for health system assessment [61], numerous strategies, such as the incorporation of low-cost HPV testing, the development of context-relevant screening and management algorithms for population-based cervical cancer screening, the utilization of existing programs and human resources by task shifting, and the enhancement of competencies of current human resources through thorough training, can help mitigate these barriers to some extent. Gupta et al. emphasized that strong political will, judicious financial allocation, and strategic communication are indispensable for the effective implementation of cancer prevention programs in settings with limited resources [62]. Since the DOST model is designed to map all potential determinants of screening uptake, it plots health system-related factors alongside beneficiary barriers, providing a holistic view, capturing both ‘beneficiary-related’ and ‘health system’ determinants.

### Beneficiary-related factors

All factors related to the beneficiary—such as sociodemographic profile, literacy, health literacy, and individual beliefs, as well as the beliefs of significant others (e.g., partner, family)—that influence the decision to undergo screening are categorized under ‘beneficiary-related factors’ in the DOST model (displayed in the yellow box in Fig. 2). These factors may include individual non-modifiable characteristics, such as sociodemographic profile, or modifiable attributes, such as beliefs and perceptions. Overall, they highlight the barriers beneficiaries face when attempting to utilize existing screening services and are a central focus of the model.

To provide clarity, beneficiary-related factors are sub-categorized as follows:

1. **Individual Factors (Factors A, B, C, D):** These factors represent the individual’s attributes and beliefs, encompassing both non-modifiable and modifiable characteristics mentioned earlier. These intrinsic factors directly shape the individual’s decision-making process regarding screening.
2. **External Factors (Factor E):** These factors encompass influences from the individual’s social context, such as family, community, or societal norms. For example, poor knowledge or negative beliefs held by a significant person (e.g., a partner or friend) may influence an individual’s beliefs, leading to a negative attitude toward screening.

This distinction between individual and external factors ensures that the model captures both internal and contextual determinants of screening behavior. By explicitly mapping these interactions, the DOST model allows implementers to target both individual barriers (e.g., knowledge gaps, false beliefs) and external influences (e.g., social support or community norms) in their interventions.

#### Factor A

##### INDIVIDUAL NON-MODIFIABLE FACTORS

Individual non-modifiable factorsepresent the **(a) sociodemographic profile**, such as age, financial status, and health insurance status; **(b) social identity** (role in family or community as a social or financial provider, decision maker, etc.); **(c) health-seeking behavior of individuals** [44] (routine response to symptoms, routine asymptomatic health check-up); and **(d) health literacy**, such as an individual’s ability to access, understand, appraise, and apply the information during the life course for health care, disease prevention or health promotion [63]. These factors are known to significantly influence the decision to undergo screening.

#### Factor B

##### KNOWLEDGE AND AWARENESS

The knowledge and awareness that individuals possess **about the (e) disease and screening** and **(f) information about an existing screening program** or services can influence their decission to undergo screening.

Specific aspects such as knowledge and awareness about disease (awareness of the disease, etiology, risk factors, nature/characteristics, warning signs, awareness of need of screening irrespective of symptoms), knowledge and awareness about screening (e.g., screening tests, methods, immediate consequences of screening both physical and screening reports) and awareness of existing screening services/programs (awareness that the test is available, where it is available, the cost of the test, the time required to undergo the test or obtain results) are vital for decision making. The individuals can obtain this information from various sources. Passive information can be obtained from general health awareness, such as media, government health education materials, friends, etc., or it can be actively sought by individuals from health practitioners or from various sources due resulting from increased interest on the topic due to presence of similar symptoms in them or among their significant relatives. The ‘knowledge and awareness’ factor is separated from others to list vital reasons for false beliefs and because they are or can usually be focused on in intervention studies.

#### Factor C

##### PERCEIVED FACTORS

Perceived factors are the beliefs and perceptions of individuals. They are concerned with various aspects, including **(g) perceived susceptibility** to the disease (i.e., beliefs about the likelihood of developing a disease or condition) [45]; for example, a woman must believe that there is a possibility of developing breast or cervical cancer before she will be interested in obtaining a mammogram. (**h) Perceived disease severity**, i.e., feelings about the seriousness of contracting an illness or of leaving it untreated, includes evaluations of both medical and clinical consequences [45]. **(i) Affect** (feelings of fear, embarrassment, anxiety related to the disease and its outcome; feelings of fear, embarrassment, anxiety related to screening process or the screening test and its outcome). **(j) Perceived benefits**, i.e., the benefit of screening/early detection and treatment (physical benefits such as improved survival rate compared with non-screening if detected), psychological benefits (peace of mind), social benefits (improved interpersonal relations), spiritual benefits (influence on spiritual beliefs), and financial benefits (prevents treatment costs of late detection of cancer).

**Perceived barriers** include those beliefs that act as barriers **(k) Perceived barriers** can be false or incorrect thoughts that are the result of incomplete ‘knowledge and awareness’, the factor mentioned (i.e., false thoughts/cognition related to the etiology of the disease, risk factors, nature/characteristics, warning signs, management, and treatment, etc.), false thoughts/cognition related to the outcome/impact of disease on one’s physical health, mental health, social relations, and spiritual or financial well-being; false thoughts/cognition related to the outcome/impact of screening on one’s physical health, mental health, social, spiritual or financial condition. These perceived barriers are **highly modifiable** through educational interventions, which aim to address and correct the underlying misconceptions.

On the other hand, perceived barriers can also be concerned with ‘**real harms’** associated with screening (e.g., discomfort during procedures, minor risks, or anxiety). These are acknowledged in the DOST model under systemic acceptability factors, within health system-related factors, and can be addressed by ensuring minimally invasive and painless procedures, offering support for managing anxiety, and providing clear communication about both benefits and risks to facilitate informed decision-making.

**(l) perceived self-efficacy** (i.e., the belief that one is capable of seeking and undergoing screening tests despite existing difficulties or challenges) [64].

#### Factor D

##### PERCEIVED STRUCTURAL AND COGNITIVE HEALTH SYSTEM-RELATED FACTORS

The Factor D includes **(m) perceived structural barriers** such as distance, time and cost involved in obtaining a screen test. For example, an individual thinking of such a screening test perceives a long distance to the hospital, the unavailability of transport, inconvenient screening hours, expensive screening, etc., as barriers. It also includes**(n) cognitive barriers** related to health system services (e.g., beliefs about provider attitudes or quality of care; lack of faith in the system due to a bad experience or information obtained; or even emotions such as fear, embarrassment, or anxiety caused by false beliefs about the health system).

Although perceived structural and health system-related barriers may appear to overlap, they are distinct in this model. Perceived structural barriers are subjective obstacles that individuals believe prevent them from accessing screening, regardless of whether these barriers exist in reality. For example, a woman may perceive that the hospital is too far away or believe that no transportation is available without actually knowing if transport services exist. In contrast, health system barriers refer to actual obstacles within the healthcare system, such as a lack of screening services at health centers due to staff shortages, inadequate infrastructure, or poor service quality, which result from systemic resource constraints or organizational inefficiencies. Although there is no universally defined cutoff guidelines for human resource or distance for determining screening accessibility, its feasibility depends on the specific implementation context. For example geopgraphical accessibility can depend on terrain, transport availability, and population distribution.

#### Factor E

##### SOCIAL FACTORS

Social factors in an individual’s context can influence the decision to undergo screening. These include (**o) the social influence of significant people** (knowledge, awareness, beliefs of partners, family and friends) and **(p) social influences from communities and groups** (e.g., norms or beliefs of the local community, culture, or tradition).

All the above factors (‘A,’ ‘B,’ ‘C,’ ‘D,’ and ‘E’) can shape ‘attitude’ (feeling that screening is not required or unnecessary) [65], ‘motivation’ (acting as facilitators), or ‘self-efficacy’-a perceived sense of ability to undergo screening.

**‘Systemic Acceptability’ v**s **‘Individual Acceptability’**: In the **DOST** model, acceptability is conceptualized in terms of two dimensions: **systemic acceptability** and **individual acceptability**. Factors categorized under **C (Perceived Factors)**,** D (Perceived Structural and Cognitive Health System-Related Factors)**, and **E (Social Factors)** influence an individual’s decision to accept screening, and can influence **individual acceptability**. Individual acceptability is shaped by **Knowledge and Awareness**, **Social Influences**, **Beliefs**, and **Emotions.** This aligns with **Sekhon’s Theoretical Framework of Acceptability (TFA)**, which conceptualizes acceptability primarily as an individual factor, influenced by **affect**,** self-efficacy**,** knowledge**, and **beliefs**. In contrast, **‘Acceptability’ categorized under ‘Health System-Related Factors’ represents systemic acceptability.** Systemic acceptability refers to the logistical and operational design of screening programs and includes factors such as **user-friendliness**,** painless procedures**, and **convenient access.** These are elements that implementers must address to make screening programs both attractive and accessible to beneficiaries. Conceptualizing acceptability as two dimensions reflects the interplay between how a screening program is **organized** (system-level) and how it is **perceived** (individual-level). This dual perspective provides implementers with actionable insights to design user-friendly screening procedures and programs while simultaneously addressing individual-level factors such as beliefs through educational campaigns and community engagement initiatives.

## Discussion

Previous research has extensively investigated a variety of factors that influence screening uptake. Evaluating all potential impediments in a specific context can be intricate, but employing a consolidated model that methodically categorizes these barriers simplifies the process, providing implementation researchers and program managers with a more comprehensive understanding of these barriers and the underlying beliefs that give rise to them.

While traditional models highlight broader domains such as susceptibility, severity, and motivation, they primarily reflect mediating variables [33] that influence the decision to undergo screening. Poor knowledge and awareness can lead to false beliefs, which in turn evoke emotions such as fear or confidenc, anxiety or reassurance. These emotions impact perceived susceptibility and severity. For example, fear and anxiety can negatively impact perceived susceptibility or perceived severity leading to a diminished perception of benefits and, consequently, poor motivation and attitudes toward screening. Therefore, to effectively address screening uptake, it is essential to target these root causes—beliefs and knowledge—to mitigate the emotional and cognitive barriers influencing the decision to undergo screening.

Identifying and mitigating poor knowledge or awareness and false beliefs arising from inadequate knowledge can enhance motivation and capabilities or alleviate barriers to improve screening uptake. In the context of formative research, tools such as the Cancer Awareness Measure (CAM) [66] or the ABC measure of cancer awareness and beliefs [67] have been used to assess awareness of cancer risk factors and warning signs, which can help assess knowledge that leads to beliefs. However, they do not explain the role of awareness about screening benefits or explore the general care-seeking attitudes or behaviors of participants, which can significantly explain screening uptake and studies have shown that the BCAM-Breast Cancer Awareness Measure tool is basic and rudimentary [66, 67]. 

In intervention research, Hungerford’s behavior change theory [68] emphasizes that modifying beliefs through education can significantly improve attitudes toward screening uptake. To address psychosocial barriers effectively, it is crucial to provide clear and comprehensive information, which can be termed ‘necessary information’ and is essential for instilling correct beliefs, potentially leading to enriched emotional responses that influence an individual’s decision to undergo screening. The DOST model, with its extensive mapping of psycho-social determinants, not only highlights topics to explore for false beliefs but also serves as a practical guide for formulating ‘necessary information’ required to educate and correct misconceptions. This approach ensures a thorough understanding and addresses all relevant barriers, making it highly actionable for designing interventions.

The DOST model domains are built upon traditional health behavior models, adopting a socioecological approach to categorize these domains effectively. It also categorizes the domains into simpler subdomains that were compared with barriers identified through empirical research that reflect real-world barriers, which makes it easy to understand for implementers and program managers, even those without an extensive background in health behavior theory. While primarily focused on psychosocial factors, DOST employs a socioecological model to incorporate a ‘systems approach,’ effectively including health system and external factors. This ensures a holistic view, integrating influences at multiple levels—individual, interpersonal, societal, and organizational while also offering an easy-to-understand, actionable framework that guides investigators and implementers in instilling accurate beliefs.

The DOST model is an amalgamation of traditional health behavior models and the socioecological model, enhancing their utility by combining complementary elements. This integrative approach provides both theoretical depth and practical insights for effectively addressing barriers to cancer screening uptake. By combining complementary elements, it explains a larger portion of the variance in screening intentions compared to using individual models alone. Additionally, integrating theoretical insights with empirical observations provides a nuanced understanding of barriers to cancer screening uptake and offers practical solutions for addressing these barriers in real-world settings.

Although the DOST model was developed with cervical cancer screening as a reference, its comprehensive framework enables broader application to other cancer screening programs. Screening uptake for breast, prostate or colorectal cancer screening, for instance, are also affected by psychosocial and cultural barriers such as fear, embarrassment, and misinformation. The socio-ecological categorization of barriers in the DOST model ensures that it is versatile enough to address these challenges while remaining adaptable to specific contextual differences. By providing a systematic mapping of barriers, the DOST model supports the design of targeted interventions, educational programs, and policy measures aimed at improving screening uptake across a range of cancers.

Future research will need to establish the DOST model as a robust enhancement tool, effectively bridging theoretical insights with practical applications in the design of barrier assessments and educational interventions. By tailoring the framework to specific cultural contexts, we can move from theoretical evidence to real-world implementation to address the'Health system factors' and'Beneficiary related-factors' that act as barriers.

### Implications

Considering the diverse needs and barriers faced by different stakeholders, the DOST model can serve specific purposes to effectively enhance cancer screening uptake. The Table 2 provides tailored implications for researchers, program managers, and policymakers, illustrating how the DOST model can be effectively utilized to enhance screening uptake.

**Table 2:** Implications of DOST model

Target audience	Implications*	
Researchers	• Barrier assessment: The DOST model can be used for formative research or contextual analysis to systematically assess barriers to screening uptake. It serves as a guide for data collection from screening program beneficiaries by delineating all potential barriers grounded in theoretical models, providing a clear outline of critical factors that significantly influence decision-making process to undergo screening. • Intervention research: For intervention research, targeted interventions can mitigate specific barriers, but educational interventions should cover all relevant aspects listed in Table 1, including communicating screening benefits and addressing barriers. Using the DOST model as a guide can help draft educational messages that address the relevant topics needed to educate women and mitigate false beliefs hindering screening uptake. These interventions must be culturally tailored, context-specific, and simplified to suit the characteristics of the target individuals.	
Program Managers	• Holistic approach: Unlike many interventions that emphasize only education and awareness, the DOST model uniquely addresses ‘modifiable barriers,’ such as false beliefs. These barriers can be mitigated through targeted information and education, making the model highly effective for developing educational materials to promote screening. By providing individuals with comprehensive information on the benefits and potential harms of screening, the model ensures they are equipped to make informed decisions about participation. Additionally, the DOST model offers a comprehensive framework for understanding external barriers to cancer screening uptake and their influence on an individual’s decision to undergo screening. This approach encourages program managers to design programs that include not just the beneficiaries but also their influencers, such as family members and the community. • Establishing effectiveness of educational informative materials through empirical evidence is vital, however, basic information materials promoting screening for cancer, must contain all the components mentioned in Table 1 to facilitate decision making.	
Policymakers	• Policy and health system factors: Various political factors and the availability of health system resources significantly influence the establishment of screening services. While the DOST model maps all determinants of screening uptake through beneficiary’s lens, ensuring the availability of affordable and accessible screening is vital before promoting screening uptake. Guidelines for screening, referrals, and the involvement of key community actors, along with accessible screening clinics and a follow-up system, can improve screening rates, early diagnosis, and treatment. • Reviewing educational materials and methods: Most educational materials provide basic information but may not fully address knowledge gaps or mitigate false beliefs. Educational materials based on health behavior models like DOST should be thoroughly reviewed to ensure they effectively promote screening sharing screening benefits and potential risks. Additionally, reliable information delivery is crucial to ensure that all educational content is communicated with fidelity. • Using model to promote screening of other cancers: Originally designed for cervical cancer screening, the DOST model is adaptable for assessing barriers to other types of cancer screenings, provided it adheres to fundamental principles of cancer screening [69]	

*It is vital to understand the ‘considerations of model use listed in Table 3 for its effective application

The foundational premise of the DOST model is the fact that individuals often do not perceive the necessity for screening in the absence of symptoms. They operate under the assumption that no apparent ailment means no need for action, unlike when symptoms manifest which trigger a response. Additionally, screening procedures, particularly those involving private body areas, may deviate from common practice and can evoke physical or psychological discomfort. These factors can render the idea of screening unfavorable unless the perceived benefits significantly outweigh the discomforts and risks. This highlights the critical need for heightened awareness and understanding of the benefits of screening, emphasizing the importance of educational interventions to address these misconceptions. The DOST comprehensive framework enables a broader application to other cancer screening programs. This gives the model scope to be applied or adapted for identifying determinants in other cancer screenings.

It is crucial to recognize that the barriers and determinants identified are not exhaustive. To ensure a thorough understanding and effective application of the model, specific considerations and limitations of model use are outlined in Table 3. These considerations are vital for maximizing the impact and effective application of the model.

**Table 3:** Key considerations for applying the ‘DOST’ model

Area	Key Considerations for DOST model application
Context and applicability	• Universal Applicability: Since the determinants are based on universally applicable health behavior models DOST is applicable across different contexts i.e., it can be used for formative research or educational interventions, serving as a guide to consider all commonly evident determinants within the population. However, the existence of barriers or facilitators may vary across different contexts and populations. • Combining educational approach with targeted interventions: In the context of intervention research, targeted interventions like reminders [70], ‘mobile screening’ [71], navigation assistance [72] can mitigate accessibility and self-testing can improve acceptability, involving families and communities can improve social support (Factor E) [73]. Combining such interventions with theory-based models like DOST can facilitate screening uptake. Without necessary information even the targeted interventions may fail to provide expected outcome.
Availability, Affordability and Accessibility before Acceptability	• Ensuring Screening Availability (Controlling Health-System Barriers): The DOST model comprehensively maps all potential psychosocial barriers that deter screening uptake. It is crucial to ensure that the ‘health system barriers’ listed in the model are mitigated, i.e., screening is available for free or at an affordable cost and accessible at convenient locations and times. This is preliminary to diving deeper into beneficiary-related barriers that explain non-uptake despite available services.
Whole or Fragmented Model Application	• The utilization of the model’s integrated components—or specific components thereof—is contingent upon the researcher’s objectives. Specific components of the DOST model can be selectively targeted based on the prevalent barriers within a given context. Alternatively, a holistic approach can be adopted, embracing all components. While the outcome of interest is to improve screening attitude, intention, or uptake, considering that any of these psychological determinants can potentially serve as barriers to uptake, utilizing the entire model to conduct a comprehensive assessment of all the listed barriers is recommended.
‘Universal precautions’ principle Vs Targeted intervention	• Applying Universal precautions principle: Educational messages should be clear, culturally acceptable, and simple to understand. Ideally, they should address all necessary elements to prevent important topics from being overlooked, particularly when reluctance exists to focus on a single barrier, which is why, DOST model exceptionally apt for program promotion, to ensure covering all information while promoting awareness. • Developing Targeted materials: A stepwise approach for assessment and mitigation is another effective method. The first step involves identifying determinants using barrier assessment tools based on the DOST model to evaluate the most influential predictors of screening uptake. This step provides clear insights into the reasons for lack of interest or nonparticipation among beneficiaries. The second step focuses on designing educational materials tailored to beneficiary characteristics such as education level, cultural beliefs, and health literacy. Additionally, informing beneficiaries about accessible screening points and low- or no-cost options can help mitigate perceived structural barriers.
Practical information and cultural sensitivity	• Including practical information: When utilizing the model to inform educational content, it is essential to remember that mitigating psychological and sociocultural beliefs alone does not ensure screening uptake. Practical implementation must be informed by empirical research. There is a complex interplay of perceived ‘structural barriers’ (Factor D) and knowledge and perceptions (Factors B, C, E). Perceived structural barriers like false beliefs related to screening like -‘high cost,’ ‘time consuming procedure,’ and ‘poor knowledge about location of screening center’ must be included with cancer and screening related information. • Cultural sensitivity: The information material must suit the language and adapt to the culture and traditional beliefs of the population, while maintaining the content. Hence, development of such materials must be carefully done and validated by involving education experts, implementers and subset of the target population.
Maintaining and reporting implementation fidelity while establishing evidence on intervention effectiveness	• Intervention and implementation determinants: Empirical research is required to establish evidence on effectiveness of education materials. Most studies that report effectiveness do not provide information on materials, their content, fidelity of delivery of contents, theoretical background, or method used to deliver this information, or the conditions or the process [74]. Various factors related to an intervention, such as its complexity, guidelines, clarity, the expertise of providers, and the interest of recipients, can influence its implementation and lead to the ineffective delivery of the designed materials, making the intervention appear ineffective. Describing the intervention, the process of implementation, and the factors that influenced it is crucial. These factors include context, intervention components, implementers, type of screening offered, barriers targeted, and facilitators used. This detailed description allows readers to draw meaningful conclusions about the outcomes obtained. This recommendation extends beyond this research to general public health research, where real-life scenarios present various unexpected factors that can influence the effectiveness of intervention implementation.
Scope to assess interrelationship of determinants	• This Model does provide a list of determinants but does not explain their interrelationships. For example, understanding Interplay of Perceived ‘Structural Barriers’ (Factor D) and Knowledge and Perceptions (Factors B, C, E). The factors, such as ‘structural barriers’ like ‘high cost,’ ‘lack of time,’ and ‘poor accessibility,’ need to be mitigated by making such screening services available as mentioned earlier. However, there is also a chance that when ‘necessary’ information on disease and screening benefits based on behavior theories is provided, beneficiaries might make an effort to undergo screening, overcoming some of the structural barriers. However, further evidence is needed to determine if educational interventions based solely on the model can effectively overcome structural barriers.
Interlinked Affective Cognitive factors	• Affect related factors such as fear, anxiety, and embarrassment are often rooted incognitive beliefs and socio-cultural norms, categorized under factors B, C and D of the DOST model. These emotional responses rarely function as a standalone barrier; instead, they arise from misinformation, false beliefs and societal stigmas. For instance, fear may result from the belief that cancer is untreatable, and embarrassment may stem from stigmatizing views of reproductive health. Categorizing “Affect” separately in the DOST model simplifies identification and its importance in interventions, while acknowledge its deep interplay with belief related determinants, as evidenced in empirical research.

### Limitations

Beneficiary-related factors to screening uptake: The model is centered explicitly on the barriers faced by or perceived by beneficiaries impeding screening uptake and does not dive deeper into the challenges related to screening implementation within the health system or the determinants of intervention implementation. For application of the model to inform interventions, one must remember that the ‘health system barrier’ is delineated as a determinant of screening uptake. That is, the model can be effectively applied when health system barriers have been addressed or when a screening program is well established and when utilization remains suboptimal.

## Conclusion

Developing barrier assessment tools remains a significant challenge for implementers due to the limitations of both top-down and bottom-up approaches. While health behavior theories show promise in designing these tools and formulating educational interventions, operational complexities often hinder their real-world application. The DOST model addresses these issues by integrating a comprehensive range of potential barriers, providing a more complete understanding of the determinants of cancer screening uptake. It facilitates the theoretical application in empirical research and encourages users to consider all possible barriers within a given context.

Future research should focus on tool development and practical applications to fully reveal the model’s potential to explore screening barriers. It is recommended that, during the development of such tools, particular attention be given to assessing their construct validity to ensure they accurately measure the theoretical domains outlined in the model. The barrier assessment questionnaire currently being developed based on the DOST model may serve as a useful reference for ensuring content validity, although further validation and refinement are ongoing. While utilizing the model to mitigate the barriers, studies should focus on its utility in patient-centered care, where addressing individual and systemic barriers ensures individuals are empowered to make autonomous, informed decisions about screening participation. Such work will ultimately enhance health behavior outcomes and public health interventions. Ongoing refinement and validation of the DOST model through empirical studies will be pivotal in adapting and implementing it across diverse health contexts and domains.

## Data Availability

No datasets were generated or analysed during the current study.
